# Successful Nemolizumab Treatment of Refractory Acquired Reactive Perforating Collagenosis in a Diabetic Patient

**DOI:** 10.7759/cureus.88884

**Published:** 2025-07-28

**Authors:** Asuka Oikawa, Ken Muramatsu, Kazuki Watanabe, Chihiro Shiiya, Hideyuki Ujiie

**Affiliations:** 1 Dermatology, Hokkaido University Hospital, Sapporo, JPN

**Keywords:** acquired perforating dermatosis, acquired reactive perforating collagenosis, chronic pruritus, diabetes mellitus type 2, nemolizumab, perforating dermatosis

## Abstract

Acquired reactive perforating collagenosis (ARPC) is a rare dermatosis associated with diabetes mellitus, characterized by transepidermal elimination of collagen and intense pruritus. We report the case of a 77-year-old Japanese woman with poorly controlled diabetes who developed multiple pruritic, keratotic nodules on her chest, back, and limbs. Histopathological examination revealed full-thickness epidermal defects with prominent transepidermal elimination of thickened collagen fibers, along with dense lymphocytic and neutrophilic infiltrates in the dermis, findings consistent with ARPC. Despite improved glycemic control, her skin lesions and pruritus persisted. Treatment with subcutaneous nemolizumab (60 mg every four weeks), an anti-IL-31 receptor A monoclonal antibody, resulted in complete resolution of pruritus and marked flattening of the skin lesions within two months. This case highlights the potential role of IL-31-targeted therapy in managing refractory ARPC. The clinical improvement observed with nemolizumab suggests it may be a promising therapeutic option in selected cases; however, further studies are warranted to confirm its efficacy.

## Introduction

Acquired reactive perforating collagenosis (ARPC) is a rare type of perforating dermatosis, clinically characterized by multiple keratotic papules with a central adherent keratinous plug, and histopathologically by transepidermal elimination of collagen fibers. It is commonly associated with diabetes mellitus and chronic renal failure and is frequently accompanied by severe pruritus. Although the precise etiology of ARPC remains unclear, superficial microtrauma resulting from pruritus and repetitive scratching in predisposed individuals is considered a major trigger in its pathogenesis [1]. Despite the use of various treatments - including corticosteroids, antihistamines, and phototherapy - the lack of effective therapies and the unknown etiology make ARPC particularly challenging to manage [2,3].

Recent evidence suggests a potential role of type 2 inflammation and pruritogenic cytokines, particularly IL-31, in the pathophysiology of ARPC [3]. IL-31 is a cytokine primarily produced by activated Th2 cells and is known to play a central role in chronic pruritus. It exerts its effects through the IL-31 receptor A (IL-31RA), which is expressed on keratinocytes, peripheral sensory neurons, and immune cells. Nemolizumab, a humanized monoclonal antibody targeting IL-31RA, has demonstrated efficacy in treating prurigo nodularis. However, its application in ARPC remains scarcely documented. Here, we report the first known case of diabetic ARPC unresponsive to standard therapies and improved glycemic control, which was successfully treated with nemolizumab.

## Case presentation

**Figure 1:**
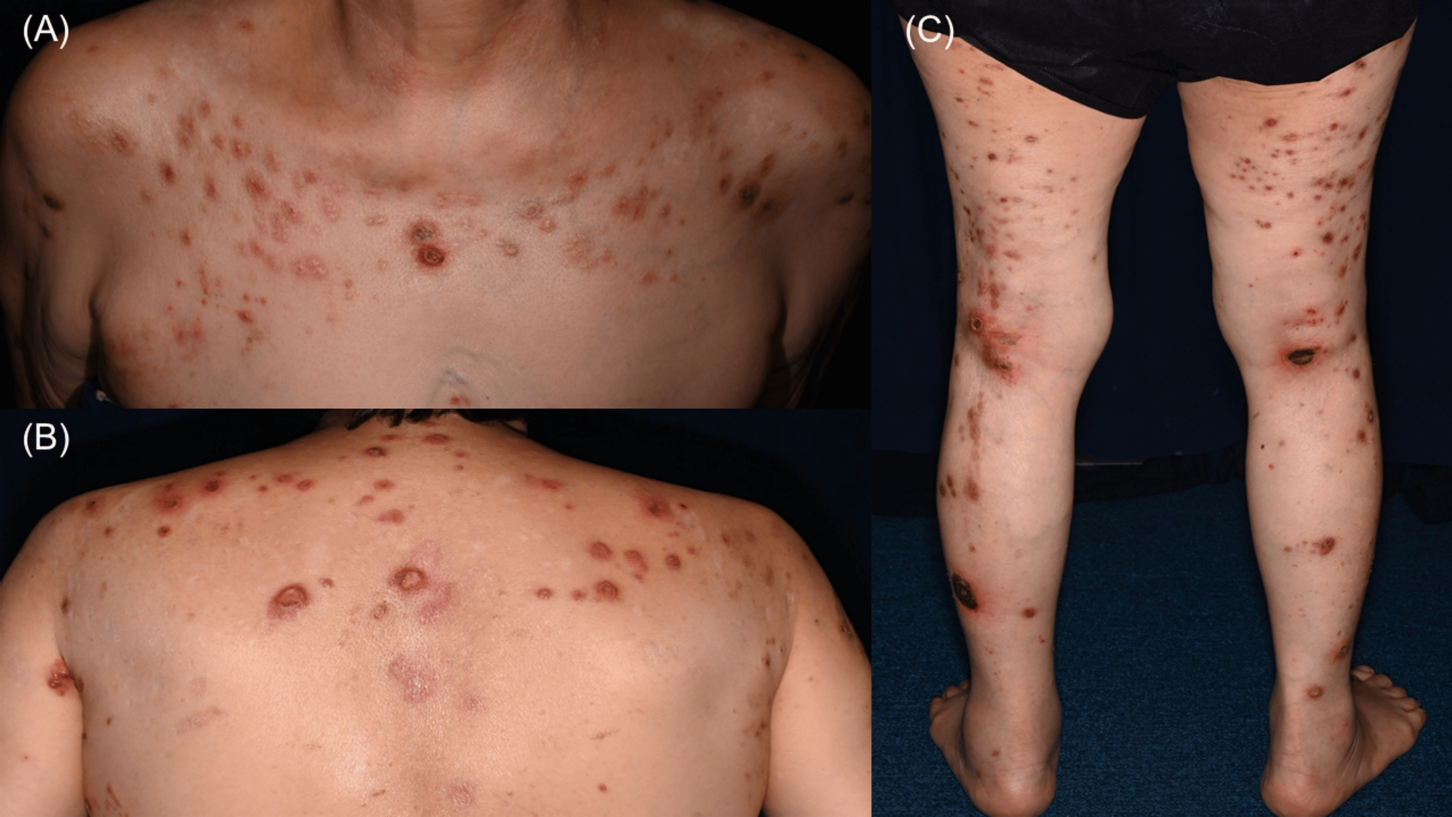
Umbilicated papulonodular lesions with brown crusts and ulcers before treatment with nemolizumab (A) Anterior chest. (B) Upper back. (C) Posterior lower limbs.

A 77-year-old Japanese woman with poorly controlled diabetes mellitus presented with pruritic skin lesions that had first appeared approximately eight years prior to her initial visit. Two years before presenting to our department, she had been prescribed topical corticosteroids and oral antihistamines; however, her skin condition progressively worsened, prompting her to seek further evaluation. Physical examination revealed firm, keratotic nodules with central plugs on her chest, back, and limbs (Figure 1A, 1B, 1C), and her itch numerical rating scale (NRS) score was 8.

**Figure 2:**
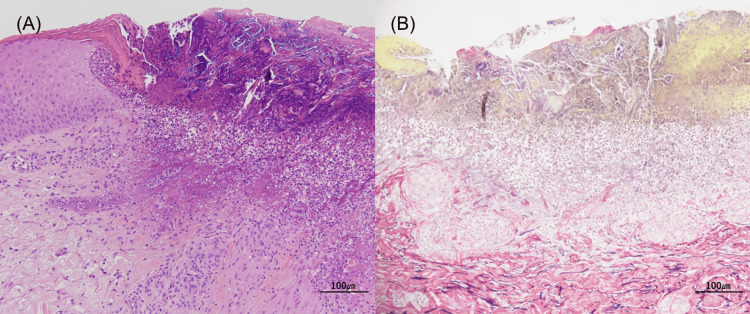
Skin biopsy specimen from the back (A) A well-circumscribed necrotic area with a keratotic plug (H&E stain; original magnification ×40). (B) Degenerated collagen bundles extending from the upper dermis to the epidermis (Elastica-Van Gieson stain; original magnification ×100).

A skin biopsy demonstrated a full-thickness epidermal defect at the center of the lesion, where the epidermis had been replaced by basophilic degenerated tissue, forming an ulcer. Thick collagen fibers were observed protruding from the dermis into the ulcerated area, accompanied by neutrophilic and lymphocytic infiltration extending into the dermis (Figure 2A, 2B).

Laboratory tests revealed normal renal and liver function (Table 1). Based on these findings, a diagnosis of ARPC was made. Her HbA1c level had increased rapidly from 7.8% to 9.1% over four months (Table 2), prompting the initiation of intensive insulin therapy. Although glycemic control improved, with HbA1c decreasing from 9.1% to 7.1% (Table 2), her skin condition showed minimal improvement. As a result, subcutaneous nemolizumab (60 mg every four weeks) was initiated two months after achieving better glycemic control.

**Table 1:** Laboratory test results at the initial visit

Test	Result	Reference range
Hemoglobin (g/dL)	10.8	12.0-16.0
White blood cell count (/µL)	5,900	3,300-8,600
Platelet count (×10⁴/µL)	22.1	15.8-34.8
Aspartate aminotransferase (IU/L)	18	13-30
Alanine aminotransferase (IU/L)	13	7-23
Total bilirubin (mg/dL)	1.1	0.4-1.5
Creatinine (mg/dL)	0.67	0.46-0.79
Blood urea nitrogen (mg/dL)	19	8-20

**Table 2:** Temporal changes in HbA1c levels in relation to diabetes treatment

Test	-4 months	Initial visit	+4 months (post-intensified therapy)	Reference range
HbA1c (%)	7.8	9.1	7.1	4.9-6.0

**Figure 3:**
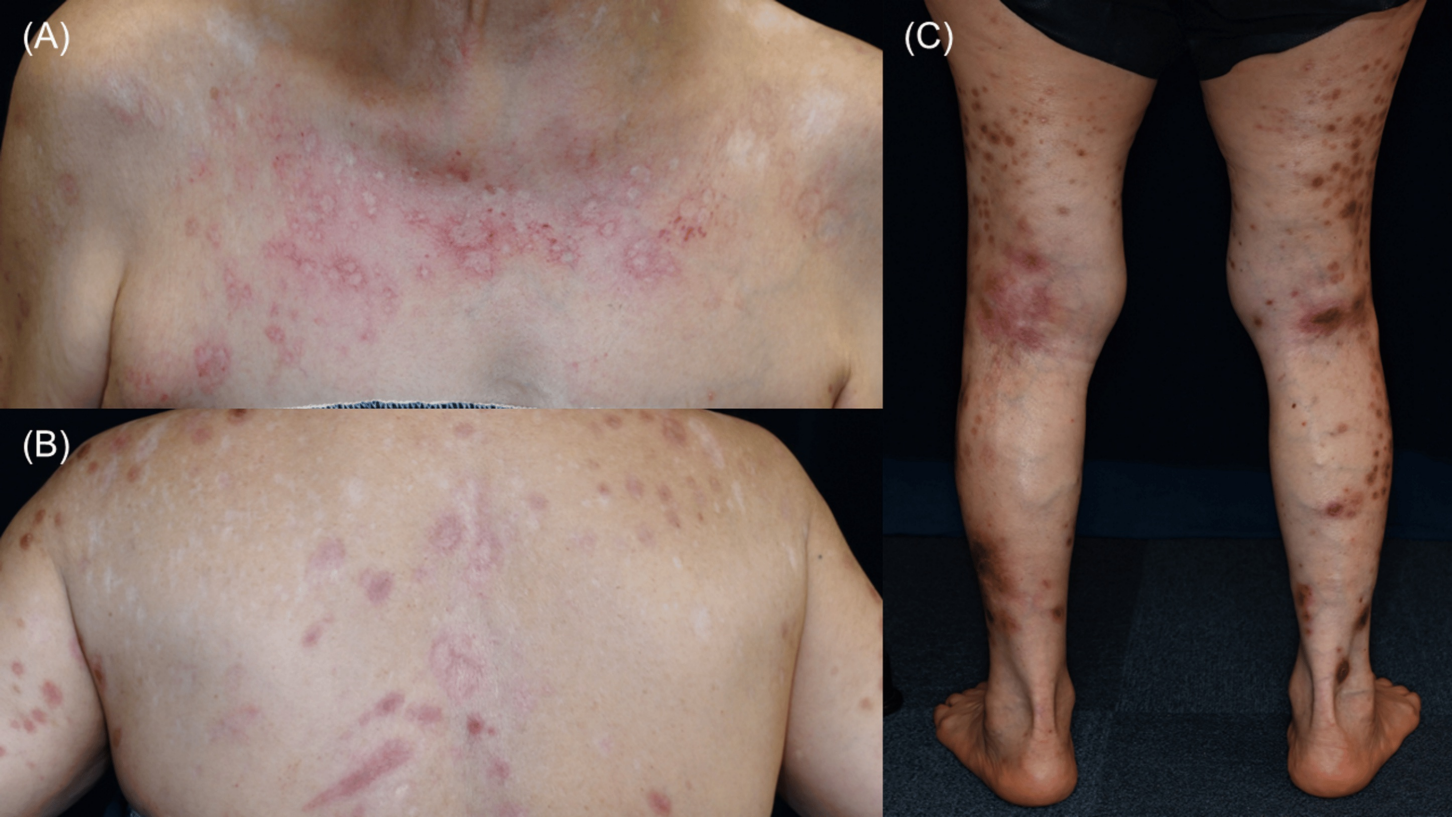
Flattened skin lesions with postinflammatory erythema or hyperpigmentation and areas of central hypopigmentation after two months of nemolizumab treatment (A) Anterior chest. (B) Upper back. (C) Posterior lower limbs.

Two months later, her NRS score had improved to 0, and the skin lesions had almost completely flattened (Figure 3A, 3B, 3C). However, at the patient’s request, treatment was discontinued after the second dose. Four months after the final administration, keratotic erythematous plaques reappeared on the occipital region.

## Discussion

ARPC is a perforating dermatosis characterized by transepidermal elimination of degenerated dermal components. Clinically, it commonly presents as multiple keratotic papules with central umbilication, often accompanied by severe pruritus. Due to its unclear etiology, management remains challenging. Although various treatments such as topical and systemic corticosteroids, narrowband ultraviolet B therapy, and antihistamines have been employed, no standardized therapeutic strategy has been established [3]. Mild surface trauma resulting from pruritus and scratching is considered a key trigger in predisposed individuals [1].

IL-4, IL-13, and IL-31 are known to activate sensory neurons that mediate pruritus [2]. In ARPC, there is dermal infiltration by CD3⁺ T cells with a Th2 phenotype, along with significant increases in IL-4 and IL-13 expression [3]. Dupilumab, a humanized monoclonal antibody targeting the IL-4 receptor alpha subunit and inhibiting IL-4 and IL-13 signaling, has shown superior efficacy compared to conventional treatments in ARPC patients [3].

The infiltration of Th2 cells in the dermis of ARPC patients further supports the hypothesis that IL-31 may also play a critical role in the pruritic pathogenesis of the disease [3]. Kawakami et al. [1] reported markedly increased expression of μ-opioid receptors in keratinocytes within ARPC lesions. Since activation of these receptors is generally associated with pruritus, further experiments were conducted and revealed strong IL-31 expression in keratinocytes overexpressing μ-opioid receptors. These findings suggest a possible link between IL-31 and ARPC-associated pruritus.

Given the central role of pruritus in our patient and the suspected involvement of IL-31, treatment with nemolizumab - a humanized monoclonal antibody against IL-31RA - was considered a rational therapeutic choice. Moreover, unlike dupilumab, which requires subcutaneous administration every two weeks, nemolizumab can be administered once every four weeks in a hospital setting, contributing to improved compliance in this elderly patient. We therefore initiated nemolizumab therapy, which resulted in notable improvement in both pruritus and skin lesions.

A review of the literature revealed only two previously reported cases in which nemolizumab was highly effective in treating ARPC [4,5]. To our knowledge, this is only the third reported case worldwide, and nemolizumab is not yet included in the Japanese treatment guidelines for ARPC [6]. The same applies to dupilumab, which, despite several case reports and systematic reviews demonstrating efficacy, is also not currently listed in the guidelines [7]. We believe this case provides valuable evidence supporting the potential inclusion of IL-31-targeted therapy in future treatment recommendations, particularly if additional cases with favorable outcomes are reported.

## Conclusions

This case highlights the potential utility of nemolizumab in the treatment of refractory ARPC associated with diabetes. Given the proposed role of IL-31 in the pruritic pathogenesis of ARPC, IL-31-targeted therapy may represent a promising option for patients who are unresponsive to conventional treatment. However, as this observation is based on a single case, it should be interpreted with caution. Further controlled studies with longer follow-up are warranted to confirm efficacy and establish treatment guidelines.

## References

[1] Kawakami T, Ikeda T, Yokoyama K, Dong Y (2023). μ-opioid receptor overexpression in acquired reactive perforating collagenosis associated with IL-31. J Dermatol Sci.

[2] Zhang LW, Wu J, Chen T (2024). Comment on 'Perforating dermatosis in a patient on haemodialysis successfully treated with nemolizumab'. Clin Exp Dermatol.

[3] Liu B, Wu Y, Wu X, Zhong X, Xue R, Zhang Z (2023). Dupilumab improve acquired reactive perforating collagenosis characterized by type 2 inflammation. Front Immunol.

[4] Ohmori S, Sawada Y (2023). Perforating dermatosis in a patient on haemodialysis successfully treated with nemolizumab. Clin Exp Dermatol.

[5] Yamada K, Baba A, Kanekura T (2024). A giant variant of acquired reactive perforating collagenosis successfully treated with nemolizumab. J Eur Acad Dermatol Venereol.

[6] Kawakami T, Akiyama M, Ishida-Yamamoto A, Nakano H, Mitoma C, Yoneda K, Suga Y (2020). Clinical practice guide for the treatment of perforating dermatosis. J Dermatol.

[7] Hu QiJ, Chen JJ, Yao X (2023). Systematic review and meta-analysis literature-based clinical retrospective analysis of acquired reactive perforating collagenosis. Int J Dermatol Venereol.

